# Supplementary material for: Swarm Learning for decentralized and confidential clinical machine learning
Source: Nature. 2021 May 26;594(7862):265–70. doi: 10.1038/s41586-021-03583-3 (PMC8189907; doi:10.1038/s41586-021-03583-3)
Supplement: Supplementary file 1 — This file contains a more detailed description of Swarm Learning and the scenarios that were used for evaluation, as well as a Supplementary Discussion. [file 41586_2021_3583_MOESM1_ESM.pdf]

nature portfolio

https://doi.org/10.1038/s41586-021-03583-3

Supplementary information

Swarm Learning for decentralized and confidential clinical machine learning

In the format provided by the authors and unedited

Swarm Learning for decentralized and confidential clinical machine learning

Stefanie Warnat-Herresthal[1,2,*], Hartmut Schultze[3,*], Krishnaprasad Lingadahalli Shastry[3,*], Sathyanarayanan Manamohan[3,*], Saikat Mukherjee[3,*], Vishesh Garg[4,*], Ravi Sarveswara[3,*], Kristian Händler[1,5,*], Peter Pickkers[6,*], N. Ahmad Aziz[7,8,*], Sofia Ktena[9,*], Florian Tran[10,11], Michael Bitzer[12], Stephan Ossowski[13,14], Nicolas Casadei[13,14], Christian Herr[15], Daniel Petersheim[16], Uta Behrends[17], Fabian Kern[18], Tobias Fehlmann[18], Philipp Schommers[19], Clara Lehmann[19,20,21], Max Augustin[19,20,21], Jan Rybniker[19,20,21], Janine Altmüller[22], Neha Mishra[11], Joana P. Bernardes[11], Benjamin Krämer[23], Lorenzo Bonaguro[1,2], Jonas Schulte-Schrepping[1,2], Elena De Domenico[1,5], Christian Siever[3], Michael Kraut[1,5], Milind Desai[3], Bruno Monnet[3], Maria Saridaki[9], Charles Martin Siegel[3], Anna Drews[1,5], Melanie Nuesch-Germano[1,2], Heidi Theis[1,5], Jan Heyckendorf[23], Stefan Schreiber[10], Sarah Kim-Hellmuth[16], COVID-19 Aachen Study COVAS, Jacob Nattermann[24,25], Dirk Skowasch[26], Ingo Kurth[27], Andreas Keller[18,28], Robert Bals[15], Peter Nürnberg[22], Olaf Rieß[13,14], Philip Rosenstiel[11], Mihai G. Netea[29,30], Fabian Theis[31], Sach Mukherjee[32], Michael Backes[33], Anna C. Aschenbrenner[1,2,5,29], Thomas Ulas[1,2], German COVID-19 OMICS Initiative (DeCOI), Monique M.B. Breteler[7,34,#], Evangelos J. Giamarellos-Bourboulis[9,#], Matthijs Kox[6,#], Matthias Becker[1,5,#], Sorin Cheran[3,#], Michael S. Woodacre[3,#], Eng Lim Goh[3,#], Joachim L. Schultze[1,2,5#]

Affiliations:
1 Systems Medicine, German Center for Neurodegenerative Diseases (DZNE), Bonn, Germany
2 Genomics and Immunoregulation, Life & Medical Sciences (LIMES) Institute, University of Bonn, Bonn, Germany
3 Hewlett Packard Enterprise, Houston, Texas, USA
4 Former employee of Hewlett Packard Enterprise
5 PRECISE Platform for Single Cell Genomics and Epigenomics at German Center for Neurodegenerative Diseases (DZNE) and the University of Bonn, Bonn, Germany
6 Department of Intensive Care Medicine and Radboud Center for Infectious Diseases (RCI), Radboud University Medical Center, Nijmegen, The Netherlands
7 Population Health Sciences, German Center for Neurodegenerative Diseases (DZNE), Bonn, Germany
8 Department of Neurology, Faculty of Medicine, University of Bonn, Bonn, Germany
9 4th Department of Internal Medicine, National and Kapodistrian University of Athens, Medical School, Athens, Greece
10 Department of Internal Medicine I., Christian-Albrechts-University and University Hospital Schleswig-Holstein, Campus Kiel, Kiel, Germany
11 Institute of Clinical Molecular Biology, Christian-Albrechts-University and University Hospital Schleswig-Holstein, Campus Kiel, Kiel, Germany
12 Department of Internal Medicine I, University Hospital, University of Tübingen, Tübingen, Germany
13 Institute of Medical Genetics and Applied Genomics, University of Tübingen, Tübingen, Germany
14 NGS Competence Center Tübingen, Tübingen, Germany
15 Department of Internal Medicine V, Saarland University Hospital, Homburg, Germany
16 Department of Pediatrics, Dr. von Hauner Children's Hospital, University Hospital LMU Munich, Munich, Germany
17 Children's Hospital, Medical Faculty, Technical University Munich, Munich, Germany
18 Clinical Bioinformatics, Saarland University, Saarbrücken, Germany
19 Department I of Internal Medicine, Faculty of Medicine and University Hospital of Cologne, University of Cologne, Cologne, Germany
20 Center for Molecular Medicine Cologne (CMMC), University of Cologne, Cologne, Germany

21 German Center for Infection Research (DZIF), Partner Site Bonn-Cologne, Cologne, Germany
22 Cologne Center for Genomics and West German Genome Center, University of Cologne, Cologne, Germany
23 Department Division of Clinical Infectious Diseases, Research Center Borstel and German Center for Infection Research (DZIF), Partner Site Hamburg-Lübeck-Borstel-Riems, Borstel, Germany
24 Department of Internal Medicine I, University Hospital Bonn, Bonn, Germany
25 German Center for Infection Research (DZIF), Braunschweig, Germany
26 Department of Internal Medicine II - Cardiology/Pneumology, University of Bonn, Bonn, Germany
27 Institute of Human Genetics, Medical Faculty, RWTH Aachen University, Aachen, Germany.
28 Department of Neurology and Neurological Sciences, Stanford University School of Medicine, Stanford, USA
29 Department of Internal Medicine and Radboud Center for Infectious Diseases (RCI), Radboud University Medical Center, Nijmegen, The Netherlands
30 Immunology & Metabolism, Life and Medical Sciences (LIMES) Institute, University of Bonn, Bonn, Germany
31 Institute of Computational Biology, Helmholtz Center Munich (HMGU), Neuherberg, Germany
32 Statistics and Machine Learning, German Center for Neurodegenerative Diseases (DZNE), Bonn, Germany
33 CISPA Helmholtz Center for Information Security, Saarbrücken, Germany
34 Institute for Medical Biometry, Informatics and Epidemiology (IMBIE), Faculty of Medicine, University of Bonn, Bonn, Germany

* shared first authors
[#] shared last authors
corresponding author: joachim.schultze@dzne.de

Supplementary Information

Medicine is a prime example to illustrate the advantages of Swarm Learning (SL). Without any doubt, numerous medical features including radiograms or computed tomographies, proteomes, metagenomes or microbiomes derived from body fluids including nasal or throat swaps, blood, urine or stool are all excellently suitable medical data for the development of AI-based diagnostic or outcome prediction classifiers. We here chose to evaluate the cellular compartment of peripheral blood, either in form of peripheral blood mononuclear cells (PBMC) or whole blood-derived transcriptomes, since blood-derived transcriptomes include important information about the patients' immune response during a certain disease, which in itself is an important molecular information[35]. In other words, in addition to the use of blood-derived high-dimensional molecular features for a diagnostic or outcome classification problem, blood transcriptomes could be further utilized in the clinic to systematically characterize ongoing pathophysiology, predict patient-specific drug targets and trigger additional studies targeting defined cell types or molecular pathways, making this feature space even more attractive to answer a wide variety of medical questions[34,36]. Here, we illustrate that newly generated blood transcriptome data together with data derived from more than 16,400 samples in 127 studies combined with AI-based algorithms in a SL environment can be successfully applied in real-world scenarios to detect patients with leukemias, tuberculosis or active COVID-19 disease in an outbreak scenario across distributed datasets without the necessity to negotiate and contractualize data sharing.

Concept of Swarm Learning

SL supports different functions for parameter merging including average, weighted average, minimum, maximum, or median functions. The various merge techniques and merge frequencies enable SL to efficiently work with imbalanced and biased data. The parameter merging algorithm is executed using a blockchain smart contract thus protects it from semi-honest or dishonest participants (**Extended Data Fig.1c**). As currently developed, SL works with parametric models with finite sets of parameters, such as linear regression or neural network models. The process of learning rounds is repeated until stopping criterions are reached, which are negotiated between the Swarm nodes/members. The leader is dynamically elected using a blockchain smart contract for merging the parameters and there is no need for a central coordinator in this Swarm network (**Extended Data Fig.1b-c**). This has two

advantages; it reduces the potential for dataset reconstruction attacks as there is no central node with global models and it provides better resilience and load balancing by distributing the job of parameter merging.

For deployment of a Swarm Network, each node is provided with a software package that contains 2 containers, the Swarm Network container, and the Swarm ML container. The Swarm Network container includes 1) software to setup and initialize the Swarm Network, 2) management commands to control the Swarm Network, and 3) start/stop SL tasks. This container also encapsulates the blockchain software. The Swarm ML container includes software to support 1) decentralized training, 2) integration with ML frameworks, and 3) it exposes APIs for ML models to interact with SL. For any ML model to be decentralized and applied to SL, it needs to be modified using the Swarm API. This Swarm API is synonymously named Swarm callback API hereafter. The callback API provides options to control the SL processes. The application layer consists of the content, which are the models for the respective domain, here medicine (**Extended Data Fig.1d**), for example models for analysis of blood transcriptome data from patients with leukemias, tuberculosis and COVID-19 (**Fig.1f-k**) or radiograms (**Fig.1l**). We chose diseases as use cases that are both heterogeneous and life-threatening to illustrate the immediate medical value of SL. Collectively, SL allows for completely decentralized ML, and therefore enables a democratized, secure, confidentiality-enabling, hardware-independent and scalable machine learning environment, removing the central parameter server (custodian). It is applicable to many scenarios and domains, which we demonstrate with four medical examples.

Swarm Learning predict leukemias from peripheral blood mononuclear cells

In a first scenario, we randomly distributed samples per node as well as cases and controls unevenly at the nodes and between nodes (dataset A2) (**Fig.2b**). Sample distribution between sample sets was permuted 100 times (**Extended Data Fig.2a**) to determine the influence of individual samples on overall performance. Among the nodes, the best test results were obtained by node one (with an even distribution between cases and controls) with a mean accuracy of 97.1%, mean sensitivity of 97.8%, mean specificity of 96.5%, and a mean AUC of 0.996, albeit this node had the smallest number of overall training samples (8% of all samples). Node 2 did not produce any meaningful results, which was due to a too low ratio of cases to controls (1:99) for training. Surprisingly, node three with the largest number of samples, but

an uneven distribution (70% cases : 30% controls) did not perform as good as node one with a mean accuracy of 94.6%. Most importantly, however, when comparing accuracy values, SL outperformed each of the nodes resulting in higher test accuracy (mean accuracy 97.5%) and AUC (mean AUC 0.998) (**Fig.2b, lower panel, Supplementary Table 4**). The accuracy of SL was significantly higher ($p < 0.01$, **Supplementary Table 5**) when compared to the performance of each of the three nodes, despite the fact that information from the poorly performing node 2 was integrated. An alternative to SL would be to centralize data in cases where data can be shared, and data privacy regulations would be fulfilled. In this first scenario, the central model performed slightly better (**Extended Data Fig.2b, Supplementary Table 4**).

To test whether more evenly distributed samples at the nodes would improve individual node performance, we distributed similar numbers of samples to each of the nodes (⅓ of training samples per node) but kept case:control ratios similar to scenario 1 (**Fig.2c, Extended Data Fig.2f-g**). While there was a slight increase in test accuracy at nodes 1 and 2, node 3 performed worse with also higher variance. More importantly, SL still resulted in the best performance metrics (mean 98.5% accuracy, mean AUC 0.998) with slightly but significantly ($p<0.001$) increasing performance compared to the first scenario and accuracy of SL in this scenario was almost at par with a central model (**Extended Data Fig.2h**). Results derived from datasets A1 and A3 echoed these findings (**Extended Data Fig.2f,i-j**).

In a third scenario, we distributed the same number of samples across all three nodes, but increased potential batch effects between nodes, by siloing training samples of a particular clinical study, independently performed and published in the past, to a dedicated training node (complete workflow in **Extended Data Fig.3a**). In this scenario, cases and control ratios varied between nodes and left out samples (independent samples) from the same published studies were combined for testing at the test node. First, we tested the performance of the three nodes and SL on the global test node. Node performances were very comparable, but never reached SL results (mean 98.8% accuracy, Swarm outperformed all nodes with $p<0.001$, **Fig.2d, Extended Data Fig.3b, Supplementary Tables 4,5**). Here, the central model performed slightly better (**Extended Data Fig.3c**). Together, these results suggested that the individual nodes cannot cope with the batch effects between the nodes, which is not the case for SL. This is further substantiated by significantly improved node-internal performance, when each node is tested using node-internal independent training and test datasets (**Extended Data Fig.3d**). Comparable results were also achieved for datasets A1 and A3 (**Extended Data Fig.3e-f**).

Along these lines, we designed a real-world scenario with siloing samples from independent previous studies not only to the training nodes, but also the global test node (**Extended Data Fig.3g**). For example, three consortia with their own independent studies would build the Swarm and then test their results on independent data provided for testing by a fourth consortium. Clearly, in this scenario batch effects are further increased. Although the variance in the results was increased both at the training nodes and for SL, SL clearly outperformed the individual nodes (mean 95.3% accuracy, **Extended Data Fig.3g-h**).

In a fourth scenario, we further optimized the nodes by increasing the overall sample size at node 3 and keeping case:control ratios even at all nodes (**Extended Data Fig.4a-f**). Clearly, node performance further improved with little variance between permutations, however, even under these 'node-optimized' conditions, SL led to higher performance parameters and accuracy and AUC were at par with the central model (both AUC 0.998, **Extended Data Fig.4d**).

In a fifth scenario, we tested whether or not SL was 'immune' against the impact of the data generation procedure (microarray versus RNA-seq) (**Fig.2e, Extended Data Fig.4g-i**). We recently demonstrated that classifiers trained on data derived by one technology (e.g., microarrays) do not necessarily perform well on another (e.g., RNA-seq)[3]. To test this influence on SL, we siloed the training samples from the three different datasets (A1-A3) to one node each, e.g., dataset A1 was used for training only at node 1. We used 20% of the data (independent non-overlapping to the training data) from each dataset (A1-A3) and combined them to form the test dataset. Node 3, trained on RNA-seq data, performed poorly on the combined dataset due to the fact that two-thirds of the data in the test dataset were microarray-derived data. Nodes 1 and 2 performed reasonably well with mean accuracies of 96.0% (node 1) and 97.5% (node 2), however did not reach the test accuracy of SL (98.7%, AUC 0.999), which also indicated that SL is much more robust toward effects introduced by different data production technologies in transcriptomics (**Fig.2e, Extended Data Fig.4g-i**) and almost reached accuracy of the central model. (**Extended Data Fig.4i)**.

Since RNA-seq can be performed using numerous different techniques, we assessed the impact of different RNA-seq protocols on predictive performance and if SL could overcome these limitations. In a sixth scenario, we split the data accordingly (**Extended Data Fig.4j-k**). While samples at node 1 were sequenced with 50 bp paired end reads using an Illumina HiSeq 2500 instrument, the AML cases from node 2 were sequenced with 100 bp paired end reads on an Illumina HiSeq 2000 instrument. Library preparation was performed using TruSeq library

preparation kit in both cases. **(Extended Data Fig.4j-k**, details on all included studies are listed in **Supplementary Table 6**). We illustrate that SL outperforms the nodes with unequal distribution of the data.

Next, we repeated several of these scenarios but this time using the second most prevalent disease, acute lymphoblastic leukemia (ALL), as cases (**Extended Data Fig.5a-b** and data not shown) and demonstrated very similar results with SL outperforming the classifiers built at the nodes. Also here, samples were siloed at nodes according to their study origin. In addition, we tested how SL would perform in a test dataset with low prevalence for ALL (10%, 5% and 1%) (**Extended Data Fig.5c-e**). While node performance dropped with decreasing prevalence, SL outperformed the nodes in all measures with the strongest effect being present at the lowest prevalence.

We next extended SL to a multi-class prediction problem, identifying patients with all four major classes of leukemias (ALL, AML, CLL and CML). We split the A2 dataset into three training and one test node with different prevalence per disease and node (**Extended Data Fig.5f**). The test node was balanced for AML, ALL and CLL, but had a low prevalence of CLL. SL outperformed the nodes or was at par with the best nodes in accuracy, sensitivity and specificity for the overall prediction as well as for the prediction of the individual diseases (**Extended Data Fig.5g, Supplementary Table 4**).

Another likely scenario for SL in medicine is the usage of data from many individual smaller medical centers. To assess such a scenario, we siloed the training data in 32 smaller nodes (**Extended Data Fig.5h**). While node prediction varied between nodes due to different distributions of case and controls, SL outperformed the nodes in all measures (**Extended Data Fig.5i, Supplementary Tables 4,5).** When increasing nodes, one can also envision that additional nodes might onboard at a later time point. Such an onboarding scenario was simulated with an initial three-node Swarm with subsequent onboarding of three additional nodes during the training cycle. Interestingly, accuracy increased immediately after onboarding of the additional nodes (**Extended Data Fig.5j**). To further illustrate the applicability of SL, we assessed whether simpler models such as LASSO could also be applied to predict leukemia (**Extended Data Fig.6a**). Again, SL outperformed the nodes, however LASSO presented with higher overall variance than the DNN trained on the same data (**Extended Data Fig.6b-c**).

Collectively, these different use cases and scenarios, using real-world transcriptome data collected from 127 individual studies, illustrate that SL would not only allow data to be kept at

the place of generation and ownership, but it also outperforms every individual node in numerous scenarios, even in those with nodes included that cannot provide any meaningful classifier results, and reaches central model performance when training data are large enough.

Swarm Learning to identify patients with tuberculosis

To apply SL, we generated a dataset based on full blood transcriptomes derived by PaxGene blood collection followed by bulk RNA-seq. We also generated new blood transcriptomes and added existing studies to the dataset compiling a total of 1,999 samples from nine individual studies including 775 active and 277 latently infected Tb cases (**Fig.1i, Extended Data Fig.7a, Supplementary Table 2**). These data are more challenging, since infectious diseases show more variety due to biological differences with respect to disease severity, phase of the disease or the host response. But also, the technology itself is more variable with numerous different approaches for full blood transcriptome sample processing, library production and sequencing, which can introduce technical noise and batches between studies.

As a first scenario, we used all Tb samples (latent and active) as cases and divided Tb cases and controls evenly among the nodes (**Extended Data Fig.7a-b, Supplementary Table 1**). Similar to AML and ALL, in detecting Tb, SL outperformed the individual nodes in accuracy (mean 93.5%), sensitivity (mean 96.1%) and specificity (mean 91.0%) (**Extended Data Fig.7b**). These values were slightly better when comparing the performance to the central model (**Extended Data Fig.7b**). To increase the challenge, we decided to assess prediction of active Tb cases only. In this scenario, latently infected Tb cases are not treated as cases but rather as controls (**Extended Data Fig.7a**). For the first scenario, we kept cases and controls even at all nodes but further reduced the number of training samples (**Fig.3a**). As expected in this more challenging scenario, distinguishing active Tb from the control cohort (including latent Tb samples), overall performance (mean accuracy 89.1%, mean sensitivity 92.2%, mean specificity 86.1%) dropped, but still SL performed better than any of the individual nodes ($p<0.01$ for Swarm vs. each node, **Fig.3a, Supplementary Table 5**). To determine whether sample size impacts on prediction results in this scenario, we reduced the number of samples at each training node (1-3) by 50% but kept the ratio between cases and controls (**Extended Data Fig.7c**). Still, SL outperformed the nodes, but all statistical readouts (mean accuracy 86.4%, mean sensitivity 87.9%, mean specificity 85.0%) at all nodes and SL showed lower performance, following general observations of AI with better performance when increasing

training data[19]. Interestingly, SL was at par with the central model here (**Extended Data Fig.7c**). We next altered the scenario by dividing up the three nodes into six smaller nodes (**Fig.3b**, samples per node reduced by half in comparison to **Fig.3a**), a scenario that can be envisioned in the domain of medicine in many settings, for example, if several smaller medical centers with less cases would join efforts (**Fig.3b**). Clearly, each individual node performed worse, but for SL the results did not deteriorate (mean accuracy 89.3%, mean sensitivity 90.6%, mean specificity 88.1% with significant difference to each of the nodes in all performance measures, see **Supplementary Table 5**), again illustrating the strength of the joined learning effort, while completely respecting each individual node's data confidentiality.

Albeit aware of the fact that active Tb is rather a disease with endemic characteristics and does not tend to develop towards a rapidly spreading pandemic such as the current COVID-19 pandemic, we utilized the Tb blood transcriptomics dataset to simulate potential outbreak and epidemic scenarios to determine benefits, but also potential limitations of SL and how to address them (**Extended Data Fig. 7d-j**). The first scenario reflects a situation in which three independent regions (simulated by the nodes), would already have sufficient but different numbers of disease cases. Furthermore, cases and controls were kept even at the test node (**Fig.3c, Extended Data Fig.7d-f**). Overall, compared to the scenario described in **Fig.3a**, results for the Swarm were almost comparable (mean accuracy 89.1%, mean sensitivity 91.2%, mean specificity 87.0%, mean AUC 0.95), while the results for the node with the lowest number of cases and controls (node 2) dropped noticeable (mean accuracy 81.2%, mean sensitivity 85.1%, mean specificity 77.3%, mean AUC 0.90; **Fig.3c, Supplementary Table 4**). When reducing the prevalence at the test node by increasing the number of controls (**Extended Data Fig.7d**), this effect was even more pronounced, while the performance of SL was almost unaffected (mean accuracy 88.0%, mean AUC 0.94).

We decreased the number of cases at a second training node (node 1) (**Extended Data Fig.7e**), which clearly reduced test performance for this particular node (**Extended Data Fig. 7e**), while test performance of the Swarm was only slightly inferior to the prior scenario (mean accuracy 85.2%, mean AUC 0.94; **Supplementary Table 4**), no significant difference to the prior scenario). Only when reducing the prevalence at the test node (**Extended Data Fig.7f**), we saw a further drop in mean specificity for the Swarm (81.9%), while sensitivity stayed similarly high (90.2%) as well as AUC (0.93). Finally, we further reduced the prevalence at two training nodes (node 2: 1:10; node 3: 1:5) as well as the test node (**Extended Data Fig.7g,h**). Lowering

the prevalence during training resulted in very poor test performance at these two nodes (accuracy node 2: 67,7%, accuracy node 3: 78.7%), while specificity was high (node 2: 98.5%, node 3: 93.9%). SL showed highest accuracy (mean accuracy 87.4%) and F1 score (83.5%) but was outperformed for sensitivity by node 1 (Swarm: 80.0%, node1: 87.9%), which showed poor performance concerning specificity (Swarm: 92.4%, node1: 84.6%). Vice versa, node 2 outperformed the Swarm for specificity (98.4%), but showed very poor sensitivity (21.2%) (**Extended Data Fig.7h**). When lowering prevalence at the test node (**Extended Data Fig.7i-j**), it became clear that all performance parameters including the F1 score were more resistant for the SL compared to individual nodes. Taken together, using whole blood transcriptomes instead of PBMC and active Tb as the disease instead of leukemia, we present a second use case illustrating that SL integrating several individual nodes outperforms each node. Furthermore, we gained initial insights into the potential of SL to be utilized in a disease outbreak scenario.

Application of Swarm Learning on chest X-ray images

To further examine potential applications and data spaces, we generated a third use case and applied the SL concept to one of the largest publicly available chest X-ray datasets[32] with over 110,000 X-ray images. We included X-rays from patients with atelectasis, effusion and infiltration as the three most frequent radiological findings in this dataset as well as images without any pathological finding, which resulted in a total of 95,831 X-ray images in dataset C (**Fig.1l, Fig.3d, Methods**). In line with our previous setup, we split the data into independent training and test datasets and siloed the training data in three training nodes. We simulated different prevalences at the nodes: While node 1 had a low prevalence in infiltration, node 2 was low in cases of atelectasis and node 3 was a low-prevalence node for images classified as effusion. Also here, SL and single nodes were tested on the global test dataset. Generally, prediction on images of the class “effusion” resulted in better test performances than the prediction of atelectasis and infiltration. Most important however, SL outperformed each of the nodes in prediction of all radiological findings included ($AUC_{Atelectasis}$: 0.76, $AUC_{Effusion}$: 0.86. $AUC_{Infiltration}$: 0.68) as well as the prediction of lack of pathological finding in the control images ($AUC_{no\ finding}$: 0.81).

Identification of COVID-19

We assessed if the Swarm network of six nodes would also predict data from completely independent external nodes (E7, E8; **Extended Data Fig.9e**, **Methods**). Node performance on E7 was very variable between datasets / centers, while SL clearly outperformed all nodes in AUC (0.98) and accuracy (96.2%, **Extended Data Fig.9f**). Interestingly, when testing on E8, including 45 samples of convalescent COVID-19 patients, four patients were classified as cases by most individual nodes as well as SL, indicating that a subgroup of COVID-19 patients still seem to contain blood transcriptomes reminiscent of acute disease (data not shown), which - in light of long COVID-19 - opens additional avenues for SL. To generate a situation for which only a small number of centers (here three) would be available for SL during an early outbreak scenario, which then would be capable to provide a predictor to a fourth independent center with acute COVID-19 patients, we assessed whether the centers E1, E2 and E3 can predict patients in E4. While all nodes show high AUC and accuracy, SL shows the highest sensitivity (1.0), again illustrating the advantage of SL over individual nodes (**Extended Data Fig.9h**).

Finally, we assessed how SL would cope with biased age and sex distributions, the influence of co-infections as well as the potential to distinguish mild from severe cases (**Extended Data Fig.10**). Distributing solely male cases to node 1 and solely female cases to node 2 did not affect node prediction performance. However, SL was also here outperforming the nodes (**Extended Data Fig.10a**). When distributing only samples from patients older than 65 to node 1, younger than 65 to node 2 and both age groups to node 3, SL outperformed nodes 1 and 2 (**Extended Data Fig.10b**). Co-infections seemed to have less impact. Biased distribution with node 1 only harboring samples from patients with co-infections, node 2 only cases without co-infections and node 3 both groups results in only slightly better performance of SL (**Extended Data Fig.10c**).

Supplementary Discussion

The introduction of precision medicine based on high-resolution molecular and imaging data will heavily rely on trustworthy ML algorithms in compute environments that are characterized by high accuracy and efficiency, confidentiality-, privacy- and ethics-preserving, secure, and fault-tolerant by design[23,24]. At the same time, privacy legislation is becoming increasingly strict, as risks of cloud-based and central data-acquisition are recognized. Here, we introduce SL, which combines blockchain technology and machine learning environments organized in a Swarm network architecture with independent Swarm edge nodes that harbor local data,

compute infrastructure, and execute the shared learning models that make central data acquisition obsolete. During iterations of SL, one of the nodes is chosen to lead the iteration, which does not require a central parameter server anymore thereby restricting centralization of learned knowledge and at the same time increasing resiliency and fault tolerance. In fact, these are the most important improvements over current local, central (i.e. cloud-based) and federated computing models. Furthermore, private permissioned blockchain technology, harboring all rules of interaction between the nodes, is Swarm Learning's inherent confidentiality-enabling strategy. This technological advancement is of particular interest to medical data and could be adapted by other federated learning systems. To understand whether the concept of SL would also be characterized by high efficiency and high accuracy, we built four medical use cases including heterogeneous diseases such as leukemias, Tb and COVID-19, for which classification is a non-trivial task[13]. Further, we apply SL to two different data spaces, blood transcriptome data, which are high-dimensional data derived from blood, one of the major tissues used for diagnostic purposes in medicine, as well as X-ray imaging. First, utilizing three previously compiled datasets (A1-3) of peripheral blood mononuclear cells derived from patients with acute myeloid leukemia, we provide strong evidence that SL-based classifier generation using a well-established neural network algorithm outperforms individual nodes, even in scenarios where individual contributing Swarm nodes were performing rather poorly. Surprisingly, it was not necessary to fine tune with applying weights to individual nodes to improve overall performance of Swarm in most scenarios, indicating that access to an enlarged dataset prevails over optimization of the AI model. Future studies will address whether the combination of better models and access to enlarged datasets can further improve SL. Most striking, SL even improved performance parameters when training of individual nodes was based on technically different data, a situation that was previously shown to deteriorate classifier performance[3]. With these promising results, we generated a more challenging use case in infectious disease patients, detecting Tb based on full blood transcriptomes. Also, in this case, SL outperformed individual nodes. Furthermore, using X-rays, we illustrate that SL also performs well in a different data space.

Using Tb to simulate scenarios that could be envisioned for building blood transcriptome classifiers for patients during an outbreak situation, we further illustrate the power of SL over individual nodes. Considering the difficulty to quickly negotiate data sharing protocols or contracts during an epidemic or pandemic outbreak, we deduce from these findings that SL would be an ideal strategy for independent producer of medical data to quickly team up to

increase the power to generate robust and reliable machine learning-based disease or outcome prediction classifier without the need to share data or relocate data to central cloud storages. Further, we generated disease prediction classifiers for COVID-19 in an outbreak scenario building on knowledge that blood transcriptomes in COVID-19 are significantly altered including gene expression changes in hundreds of genes[34,36]. Here, we provide evidence that classifiers with high accuracy, sensitivity, specificity and F1 scores can be generated to identify patients with COVID-19 based on their blood transcriptomes. Since current test strategies are intended to identify every individual infected with SARS-CoV-2 (PCR tests, antigen tests), but do not provide any information concerning the disease (asymptomatic to severe courses), blood transcriptomics could be envisioned as a complementary approach identifying patients who will require further medical attention. Moreover, we illustrate the capacity of SL allowing to quickly increase the power of classifier generation even under very early outbreak scenarios with very few cases used at the training nodes, which could be e.g., collaborating hospitals in an outbreak region. Since data do not have to be shared, additional hospitals could benefit from such a system by applying the classifiers to their new patients and once classified, one could even envision an onboarding of these hospitals for an adaptive classifier improvement schema. Albeit technically feasible, we are fully aware that such scenarios require further classifier testing and confirmation, but also an assessment of how this could be integrated in existing legal and ethical regulations at different regions in the world[4,8]. Furthermore, we appreciate that other currently less expensive data might be suitable for generating classifiers to identify COVID-19 patients[6]. For example, if highly standardized clinical data would become available, SL could be used to interrogate the clinical feature space at many clinics worldwide without any need to exchange the data to develop high performance classifiers for detecting COVID-19 patients. Similarly, recently introduced AI-systems using imaging data[15,16] might be more easily scaled if many hospitals with such data could be connected via SL. Our example using a publicly available X-ray dataset clearly points towards this direction. Irrespective of these additional opportunities using other parameter spaces, we would like to suggest blood transcriptomics as a promising new alternative due to its very strong signal in COVID-19. A next step will be to determine whether blood transcriptomes taken at early time points could be used to predict severe disease courses, which might allow physicians to introduce novel treatments at an earlier time point. Furthermore, we propose to develop an international registry of blood transcriptomes that could be utilized for the development of predictive classifiers in other infectious and non-infectious diseases as well. It could be envisioned that such an SL-based learning scheme could be deployed as a permanent monitoring or early warning system

that runs by default, looking for unusual movements in molecular profiles. Collectively, SL together with transcriptomics but also other medical data is a very promising approach to democratize the use of AI among the many stakeholders in the domain of medicine while at the same time resulting in more data confidentiality, privacy, data protection and less data traffic.

Supplementary Methods

Datasets

Peripheral blood mononuclear cell (PBMC)-derived transcriptome dataset (dataset A)

We used a previously described dataset containing over 12,000 transcriptomes derived from peripheral blood mononuclear cells (PBMC), deposited at the National Center for Biotechnology Information Gene Expression Omnibus (GEO) under SuperSeries GSE122517 or via the individual SubSeries GSE122505 (dataset A1), GSE122511 (dataset A2) and GSE122515 (dataset A3). Briefly, this dataset was generated by inspection of all publicly available datasets at GEO on September 20th, 2017. Inclusion criteria were cell type (PBMCs) and species (*Homo sapiens*). Existing GEO SuperSeries were excluded to avoid duplicated samples. According to the data generation method, three datasets were established; dataset A1, generated with Affymetrix HG-U133 A microarrays (n=2,500), dataset A2 with Affymetrix HG-U133 2.0 microarrays (n=8,348), and dataset A3 with high-throughput RNA sequencing (RNA-seq) (n=1,181). Data were curated as previously described[3]. All sample information is listed in **Supplementary Table 2.**

Whole blood-derived transcriptomes for the prediction of tuberculosis (dataset B)

To establish a dataset based on whole blood transcriptomes, we generated new data from healthy controls (Rhineland Study) and combined these with previously generated data that had been deposited in Gene Expression Omnibus (GEO). We screened for transcriptome datasets derived from human whole blood samples, which were collected using the PAXgene Blood RNA System. In total, nine independent datasets were selected to be included in the present study (GSE101705 (n=44); GSE107104 (n=33), GSE112087 (n=120), GSE128078 (n=99), GSE66573 (n=14), GSE79362 (n=355), GSE84076 (n=36); GSE89403 (n=914)). The newly generated 384 whole blood samples were sampled in context of the Rhineland Study led by the

German Center for Neurodegenerative Diseases (DZNE), which is an extensive longitudinal study monitoring healthy individuals over 2 decades. Approval to undertake the Rhineland Study was obtained from the ethics committee of the University of Bonn, Medical Faculty. The study is carried out in accordance with the recommendations of the International Conference on Harmonization (ICH) Good Clinical Practice (GCP) standards (ICH-GCP). Written informed consent was obtained from all participants in accordance with the Declaration of Helsinki. Overnight fasting blood was collected from all participants, including a PAXgene® tube for RNA extraction and RNA-seq analysis. In total, dataset B contained 1999 samples from patients with active tuberculosis (n=775), latent tuberculosis (n=277), fatigue (n=55), autoimmune diseases (n=68), HIV (n=16) and controls (n=808). Sample information is listed in **Supplementary Table 2**.

X-ray dataset (dataset C)

To evaluate whether Swarm learning can also be applied to other medical data types, we included a publicly available dataset on chest X-ray images. We used the National Institutes of Health (NIH) Chest X-ray dataset available on Kaggle – https://www.kaggle.com/nih-chest-xrays/data[32]. It is one of the largest publicly available real-world anonymized chest X-ray datasets with annotations that can be used to perform clinically relevant computer-aided detection and diagnosis (CAD). The image labels are extracted from the associated radiological reports using Natural-Language-Processing (NLP) with more than 90% labelling accuracy. The study was IRB approved (personal communication by Dr. Summers, Senior Investigator, Clinical Image Processing Service, NIH CC).

The NIH Chest X-ray dataset consists of 112,120 X-ray images of size 1024 x 1024 with pathophysiological findings from 30,805 unique patients. There are in total 15 classes (14 pathophysiological findings and 1 control): atelectasis, consolidation, infiltration, pneumothorax, edema, emphysema, fibrosis, effusion, pneumonia, pleural thickening, cardiomegaly, nodule, mass, hernia, and no finding (control). It is a multi-class multi-label dataset, i.e., images can be classified as either one or more classes or only control. Images are present in 12 zip files of ~ 2-4 GB each. In our experiment, we have included the pathophysiological findings atelectasis, effusion, and infiltration as the 3 most frequent classes as well as the control images, which totals 95,831 X-ray images from the dataset.

Whole blood-derived transcriptome dataset for the prediction of COVID-19 (dataset D)

To develop classifiers based on whole blood transcriptomes able to predict COVID-19 patients we collected an additional 134 PAXgene® tubes for RNA extraction and RNA-seq analysis from patients with acute COVID-19, of which 41 samples were either collected at the Sotiria Athens General Hospital (Ethics Committee of Sotiria Athens General Hospital, IRB 23/12.08.2019) or the ATTIKON University General Hospital in Athens (Ethics Committee of ATTIKON University General Hospital, IRB 26.02.2019), Greece, and 93 whole blood samples were collected at the Intensive Care Unit of the Radboud University Medical Centre in Nijmegen, the Netherlands. The protocol was reviewed by the local ethics committee (CMO Arnhem-Nijmegen, registration no. 2016-2923) and the study was carried out in accordance with the applicable rules concerning the review of research ethics committees and informed consent in the Netherlands. All patients or legal representatives were informed about the study details and could decline to participate. COVID-19 was diagnosed by a positive SARS-CoV-2 RT-PCR test in nasopharyngeal or throat swabs and/or by typical chest CT-scan finding. Blood for RNA-seq analysis was sampled on day 0 to 11 after admission. In the cohort in Athens, blood samples from ten healthy donors who were tested negative on SARS-CoV-2 were included as controls. The newly generated samples from the COVID-19 patients and the controls from Athens were combined with dataset B (see above) to establish dataset D. As a result, in addition to the 1999 samples derived from dataset B, dataset D included further 10 healthy controls and 134 COVID-19 samples, which makes a total of 2,143 samples. Sample information is listed in **Supplementary Tables 2 and 6**.

Extension of the transcriptome dataset for the prediction of COVID-19 (dataset E1-8)

To extend the COVID-19 analysis to even more realistic scenarios, we collected additional samples from additional medical centers in Germany and generated the new dataset E. Most centers focused on sampling COVID-19 patients with an underrepresentation of controls. To reach a realistic outbreak situation (low prevalence), we kept COVID-19 cases always at the center of origin and increased the number of controls at each node by adding control samples (**Extended Data Fig.9e).**

In total, 250 PAXgene samples from patients with acute COVID-19 and 177 samples from convalescent COVID-19 patients were collected including the 93 samples from Nijmegen and the 41 samples from Athens from dataset D. The additional whole blood PAX-gene samples were derived from Kiel (COVIDOM, Ethics Committee of the University of Kiel, IRB D466/20; 41 acute COVID-19, 4 healthy controls), Saarbrücken (CORSAAR, Ethics

Committee Medical Association of the Saarland, IRB 62/20; 50 acute COVID-19), Munich (Ethics Committee of the LMU Munich, IRB 20-263; 17 acute COVID-19, 1 convalescent COVID-19, 17 healthy controls and 15 controls with other infections), Tübingen (DeCOI Host Genomes, Ethics Committee of the Medical Faculty of the University of Tübingen, IRB 286/2020B01; 45 convalescent COVID-19), Aachen (COVAS, Ethics Committee of the Medical Faculty of the Technical University Aachen, IRB 20-085; 4 acute and 12 convalescent COVID-19), Cologne (Ethics Committee of the University of Cologne, IRB 20-1187_1; 116 convalescent COVID-19). Furthermore, we collected granulocyte-derived transcriptomes from Bonn (Ethics Committee of the Medical Faculty of the University of Bonn, IRB 073/19, 134/20; 89 acute COVID-19, 8 healthy controls and 2 controls with other infectious diseases). Briefly, granulocytes were isolated from peripheral blood collected in EDTA tubes by density centrifugation, followed by red blood cell lysis, RNA extraction and library preparation for RNA-seq.

Furthermore, we collected 1,444 healthy PAXgene control samples from Saarbrücken (Ethics Committee Medical Association of the Saarland, IRB 20200597). Diagnostics testing for SARS-CoV-2 was carried out as for patients in Athens and Nijmegen. Samples from acute COVID-19 patients were collected until day 46 after admission to the hospital or during ambulant hospital visits. Some medical centers provided longitudinal samples, in which gene expression of the same patient was measured at different time points. For training, we included only the first two time points per individual. For testing also other time points were included. Samples from convalescent patients were collected at least 46 days post symptoms. Since not all medical centers could provide healthy controls, we simulated low prevalences for COVID-19 in the scenarios described here by distributing control samples from Bonn and Saarbrücken to the data derived by the other centers so that we had a total of 300 samples per dataset E1-E8 (**Extended Data Fig.9e**). Dataset E1 contained 39 acute COVID-19 patients from Athens and 261 healthy controls, dataset E2 contained 50 acute COVID-19 patients from Saarbrücken and 250 healthy controls, dataset E3 contained 70 acute COVID-19 patients from Nijmegen, as well as 144 controls with acute sepsis and 86 healthy controls, dataset E4 contained 32 acute COVID-19 patients from Kiel and 268 healthy controls, dataset E5 contained 12 acute COVID-19 samples from Munich, 272 healthy controls, 15 samples with other diseases and 1 patient with convalescent COVID-19, dataset E6 contained 4 acute COVID-19 cases from Aachen and 128 convalescent COVID-19 patients from Cologne as well as 168 healthy controls, dataset E7 contained 89 acute COVID-19 samples, 2 samples with other infectious diseases and 209 healthy controls (89 granulocyte-derived transcriptomes and 201 whole-blood transcriptomes)

and dataset E8 contained 45 convalescent COVID-19 patients and 255 healthy controls. In total 2,400 samples were included in dataset E and sample information is listed in **Supplementary Table 2**.

Application Layer and scenarios

The application layer (see also **Extended Data Fig.1d**) consists of disease models for which definitions are given, which samples are cases and which samples are controls. For example, if the classifier is supposed to detect all patients with tuberculosis (Tb), the model includes patients with latent and active tuberculosis as cases and all other samples as controls (**Extended Data Fig.7**). However, if only patients with active tuberculosis are intended to be detected as cases, the model is changed in that cases are now only patient samples derived from patients with active Tb, while samples from patients with latent Tb are now treated as controls, similar to all other non-Tb samples. In case of COVID-19, we focused on classification of acute COVID-19 patients, while samples of convalescent patients were only used at test nodes and never for training purposes. For one prediction scenario (**Extended Data Fig.10d-e**), we stratified COVID-19 patients according to their disease severity. Patients with a WHO disease severity score of 5 or higher were classified as “severe” COVID-19 patients and used as cases opposed to controls, which were COVID-19 cases with severity scores from 1-4. Further, we provide multi-class predictions for the leukemia dataset distinguishing between ALL, AML, CLL, CML, and control (**Extended Data Fig.5f-g**). Here five different labels are used. This is similarly true for the X-ray dataset, where we used the pathophysiological findings atelectasis, effusion, infiltration and no finding (normal X-rays), where several images had more than one label (**Fig.3d**). The cases and controls used for each scenario are given in the result section in more detail. For each node, classifiers are generated by applying neural networks (for description see below) with the exception of **Extended Data Fig.6b**, where we used the LASSO algorithm (for description see below). All prediction results are given in **Supplementary Table 3**.

Scenarios for prediction of leukemias

We previously demonstrated that ML on PBMC transcriptomes can be utilized to predict AML[3]. Based on this experience, we generated sample sets within three independent transcriptome datasets (dataset A1-A3, see above) to assess different scenarios in a three-node

setting for training and an independent test node only used for testing. As indicated in **Fig.2 and Extended Data Fig.2-4**, six main scenarios with varying numbers of samples per node and varying ratios between cases and controls at each node where defined. For predicting AML, all samples derived from AML patients were classified as cases, while all other samples were labeled controls. For each scenario (**Fig.2, Extended Data Fig.2-4**) and each dataset, we permuted the sample distribution 100 times, resulting in a total of 9,600 individual predictions. The different scenarios were chosen to address the influence of sample numbers per node, the case control ratio, study design-related batch effects, sequencing protocols and transcriptome technologies used on classifier performance at the nodes, but more importantly on SL performance. Furthermore, we compared SL performance to the central model, which was trained by generating a central dataset on all samples, which was then split into training and test dataset according to the sample distribution within the SL model. Lastly, we used one scenario to apply LASSO as an alternative to develop an AML classifier under SL conditions (**Extended Data Fig.6a-c**).

When predicting ALL, all samples derived from ALL patients were classified as cases and all others as controls (**Extended Data Fig.5a-e**). Furthermore, we provide a scenario for multi-class prediction (**Extended Data Fig.5f-g**).

Sample distributions for all permutations within all scenarios are listed in **Supplementary Table 1** and all used tuning parameters are listed in **Supplementary Table 8.**

Scenarios for detecting patients with active TB

In line with the experience we gained from the prediction of AML, we used dataset B to generate scenarios for the prediction of tuberculosis in various settings, again using different scenarios in a three-node setting for training and an independent test node only used for testing. In one scenario, all patients with tuberculosis (Tb) including patients with latent and active Tb were treated as cases, while all others were defined as controls (**Extended Data Fig.7b**). In all other scenarios, cases were restricted to active Tb patients' samples, while patients with latent Tb were defined as controls together with all other non-Tb samples. Here, the question to be answered was whether the classifiers can identify patients with active Tb and could distinguish them from latent Tb and other conditions.

In one scenario (**Fig.3b**), we added three additional training nodes to test dependency of classifier performance by the number of nodes. As indicated in **Fig.3a, Fig.3c and Extended Data Fig.7d**, three scenarios with varying numbers of samples per node and varying ratios between cases and controls at each node where defined. For scenarios described within **Extended Data Fig.7e** and **Extended Data Fig.7f** we tested two prevalence scenarios in the test dataset. For each scenario we permuted the sample distribution 5-10 times, resulting in a total of 325 individual predictions. To mimic an outbreak scenario, we reduced cases also at the testing nodes to determine the effects on SL performance (**Extended Data Fig.7g-j**). Sample distributions for all permutations within all scenarios are listed in **Supplementary Table 1**.

Scenarios for detecting pathophysiological findings in X-ray images

In order to generalize and see whether the SL approach would also be applicable to other data spaces, we included a scenario on publicly available chest X-ray images (**Fig.3d**). Here, we focused on the prediction of three pathophysiological findings, namely effusion, atelectasis, and infiltration. Since many images contained multiple labels, this was a multiclass-multilabel classification setup, and we report the results for each predicted class separately. We permuted the scenario 10 times.

Simulation of outbreak scenarios to detect COVID-19 patients

Based on the promising results obtained with tuberculosis, we intended to simulate classifier building and testing for the prediction of COVID-19 in a SL setting. We used dataset B and added 143 additional samples, of which 134 samples were derived from acute COVID-19 patients (see above). We applied a three-node setting for training and an independent test node only used for testing.

In one scenario (**Extended Data Fig.8a-d**), we kept cases (n=30) and controls (n=30) evenly distributed among the three training nodes and tested three different prevalence scenarios at the test node (22:25; 11:25; 1:44). In a second scenario (**Extended Data Fig.8e-g**) we changed the ratio of cases and controls at each node (node 1: 40:60, node 2: 30:70, node 3: 20:80) and tested

two prevalence scenarios at the test node (22:25; 11:25). In a third scenario (**Extended Data Fig.8h-j**) we further reduced the number of cases at the training nodes (node 1: 30:70, node 2: 20:80, node 3: 10:90) and tested two prevalence scenarios at the test node (37:50; 37:75). Next, we tested an outbreak scenario (**Fig.4a,b, Extended Data Fig.8k,l**) with very few cases at the outbreak node 1 (20:80), an early secondary node (10:90) and a later secondary node (5:95) and three prevalence scenarios at the test node (1:1, 1:2, 1:10), resulting in a total of 220 individual predictions.

To design even more realistic scenarios, we recruited further samples from a total of eight different medical centers. In a first scenario six medical centers (nodes) teamed up as the Swarm (**Fig.4c**). Each center provided 20% held-out data and contributed these data to the independent test node T. The remaining 80% of the data are used for local learning at each node and for SL. COVID-19 predictors derived by SL or individual nodes of the Swarm are then tested on the test node T. Further, we used two independent medical centers (E7, E8) as additional independent test nodes (**Extended Data Fig.9g**). As outlined above, these nodes were very different in that one provided transcriptomes from blood-derived granulocyte-enriched COVID-19 samples (different technology), the other only transcriptomes from convalescent patients (no acute cases) (**Extended Data Fig.9e**). This scenario was permuted 20 times. Second, three of the centers with acute COVID-19 cases became members of the Swarm, but for testing, a fourth external medical center is used (E4) (**Extended Data Fig.9h**). Sample distributions for all permutations within all scenarios are listed in **Supplementary Table 1**.

For all use cases and scenarios, non-overlapping training and test datasets were always guaranteed.

The Swarm Learning framework, library, distributed ML and blockchain technologies

SL builds on top of two proven technologies — distributed ML and blockchain. Distributed ML is leveraged to train a common model across multiple nodes with a subset of the data located at each node — commonly known as the data parallel paradigm in ML — though without a central parameter server. Blockchain lends the decentralized control, scalability, and fault-tolerance aspects to the Swarm Network system to enable the framework to work beyond the confines of a single enterprise.

The SL Library is a framework to enable decentralized training of ML models without sharing the data. The SL framework is designed to make it possible for a set of nodes — each node possessing some training data locally — to train a common ML model collaboratively without sharing the training data itself. This can be achieved by individual nodes sharing parameters (weights) derived from training the model on the local data. This allows local measures at the nodes to maintain confidentiality and privacy of the raw data. Importantly, in contrast to many existing federated learning models, a central parameter server is omitted in SL.

The inherent trade-off between the effectiveness of a ML system and the privacy guarantees it can offer is subject to lively ongoing research. In this paper, we therefore decided not to integrate specific data privacy mechanisms in the design for use with our SL framework, but to make it compatible with previously used underlying ML models, including models that rely on privacy-enhancing technologies such as obfuscation and anonymization. The goal was to enable their seamless integration in the SL framework. Furthermore, SL can inherit developments to further preserve privacy such as differential privacy algorithms[40], functional encryption[41], or encrypted transfer learning approaches[42]. The framework itself does not rely on a fixed central aggregator, but instead implements a dynamic selection of changing aggregators for every merge cycle by means of smart contracts. The framework moreover implements state-of-the-art security technologies (trusted execution environment, secure containment, network encryption) to protect data from direct unauthorized access. To ensure equal conditions even at the early stages of building a Swarm Network (low number of nodes), we suggest using the SL infrastructure also at its lower boundary, namely with two nodes already.

The nodes that participate in SL, register themselves with the Swarm Network implicitly using the callback API. Here, the Swarm Network interacts with other peers using blockchain for sharing parameters and for controlling the training process. On each node, a simple Swarm callback API must be used to enable the ML model with SL capacities (see also code presented below). The Swarm container must be configured to interact with the Swarm Network (network IP and port configuration). All other complexities of setting up network, registration, parameter sharing, and parameter merging are taken care of by the Swarm callback API and the Swarm Network infrastructure.

Parameters shared from all the nodes are merged to obtain a global model. Moreover, the merge process is not done by a static central coordinator or parameter server, but rather a temporary

leader chosen dynamically among the nodes is used to perform the merge, thereby making the Swarm network decentralized. This provides a far greater fault-tolerance than traditional centralized-parameter-server-based frameworks. All the nodes can perform the role of training and merging, thereby maximizing the usage of local compute. The Swarm Network implicitly controls this.

The HPE SL Library consists of two containers, the Swarm Network container, and the Swarm ML container.

The Swarm Network container includes 1) software to setup and initialize the Swarm Network, 2) management commands to control the Swarm Network, and 3) start/stop SL tasks. This container also encapsulates the blockchain software.

The Swarm ML container includes software to support 1) decentralized training, 2) integration with ML frameworks, and 3) it exposes APIs for ML models to interact with SL.

For any ML model to be applied to SL, it needs to be modified using the Swarm callback API. The callback API provides options to control the SL processes. To convert a ML program into a Swarm ML program the following steps have to be performed:

1. Import the SwarmCallback class from the Swarm Library

from swarm 'import SwarmCallback'

SwarmCallback is a custom callback class that is built on the Keras Callback class.

2. Instantiate an object of the SwarmCallback class:

swarm_callback = SwarmCallback(min_peers = <peer count>,

sync_interval = <interval>,

use_adaptive_sync = <bool>,

val_batch_size = <batch size>,

val_data = <either a (x_val, y_val) tuple or a generator>

node_weightage = <relative weightage of node's model weights>).

In this context, ‘min_peers’ specifies the minimum number of network peers required to synchronize the insights, ‘sync_interval’ specifies the number of batches after which a synchronization is performed, ‘use_adaptive_sync’ specifies whether the *adaptive sync interval* feature should be used for tuning the sync interval. This feature is turned off by default; ‘ val_batch_size’ specifies the size of each validation batch; ‘val_data’ specifies the validation dataset. It can be either a (x_val, y_val) tuple or a generator;

3. Pass the object to the list of callbacks in Keras training code: model.fit(..., callbacks = [swarm_callback]). SwarmCallback can be included along with other callbacks also:

es_callback = EarlyStopping(...);

model.fit(..., callbacks = [es_callback, swarm_callback])

The Swarm Learning architecture principles

The SL framework has two major components, 1) the Swarm ML component runs a user-defined Machine Learning algorithm, and 2) the Swarm Network component forms the Swarm Network based on a blockchain network.

The Swarm ML component is implemented as an API available for multiple popular frameworks such as TensorFlow, Keras, PyTorch. This API provides an interface that is similar to the training APIs in the native frameworks familiar to data scientists. Calling this API automatically inserts the required hooks for SL so that nodes seamlessly exchange parameters and subsequently continue the training after setting the local models to the globally merged parameters. With a few simple code changes, the entire network learns as one cohort, with all the complexities of control and data flow taking place in an automated fashion.

Within the Swarm Network component each Swarm ML component interacts with each other using the Swarm Network component’s blockchain platform to maintain global state information about the model that is being trained and to track the training progress. The Swarm Network components use this state and progress information to coordinate the working of the SL. The Swarm Network is responsible for keeping the decentralized Swarm network in a globally consistent state. The Swarm Network ensures that all operations and the corresponding state transitions are performed in a synchronous manner. Both, state and supported operations

of the system are encapsulated in a blockchain smart contract. The Swarm Network contains the logic to elect the leader of the Swarm for every synchronization, implement fault-tolerance, and self-healing mechanisms, along with signaling among nodes for commencement and completion of various phases.

The SL framework is designed to run on both commodity and high-end machines, supporting a heterogeneous set of infrastructures in the network. It can be deployed within and across data centers. We also want to mention that currently SL has some limitations, e.g., all nodes within a Swarm use the same model, every node needs to use the same ML platform and SL works only for models that can be parameterized. Further, application of differential privacy algorithms have not been formally tested in this study. However, in principle, there are no differences between current ML settings and SL when applying such methods for data privacy protection. More important, in contrast to federated learning with star topology and a centralized coordinator, SL can support multiple topologies including fully connected, mesh, star, tree and hybrid topologies. This flexibility provides multiple options to cater into different use cases.

The Swarm Learning process

SL provides a callback API to enable swift integration with multiple frameworks. This API is incorporated into the existing ML code to quickly transform a stand-alone ML node into a SL participant in a non-intrusive way. It offers a set of commands (APIs) to manage the Swarm Network and control the training.

The SL process is as follows:

The SL process begins with enrollment of nodes with the Swarm Network, which is done implicitly by the Swarm callback function when the callback is constructed. During this process, the relevant attributes of the node are stored in the blockchain ledger. This is a one-time process.

Nodes will train the local copy of the model iteratively using private data over multiple epochs. During each epoch, the node trains its local model using one or more data batches for a fixed number of iterations until a merging criterion is reached. The merging criterion is defined by

the number of batches used and can be specified by the user using the sync_interval parameter in the Swarm callback API.

At the end of every synchronization interval, when it is time to share the learnings from the individual models, one of the Swarm nodes is dynamically elected as a leader. For the Swarm networks shown in this work, the leader election logic is implemented as follows: the nodes that finish the local training checks in for the merge process. The first node to check-in is the leader. The elected leader node collects the model parameters from each peer node and merges them. The framework supports multiple merge algorithms such as mean, weighted mean, median (see below), which is selected by the Swarm initiator when setting up the Swarm. Each node then uses these merged parameters to calculate various validation metrics, such as precision, F1 score, or AUC. These results are compared against the stopping criterion and if it is found to be met, the SL process is halted. The stopping criterion for the training is configurable within the callback API (such as precision to be reached). Else the nodes use the merged parameters to start the next training batch. The merged parameters can then be used by the nodes to test new, local datasets. The SL Library uses blockchain smart contracts to define the leader election logic and the merge algorithm. The blockchain smart contracts prevents attacks from semi-honest or dishonest participants.

The Swarm Learning implementation

SL implementation is divided into these phases

(1) Initialization and onboarding. Onboarding is an offline process that involves multiple entities interested in Swarm-based ML to come together and formulate the operational and legal requirements of the decentralized system. This includes aspects such as parameter sharing agreements, arrangements to ensure node visibility across organizational boundaries of the entities, and a consensus on the expected outcomes from the model training process. Configurable parameters, such as the initial node, which is necessary to span up the network, are supplied during boot-up and the synchronization frequency among nodes are finalized. The common model to be trained is defined and if applicable a reward system is agreed upon.

(2) Installation and configuration: all the consortium members download and install the Swarm platform on their respective systems (nodes), during which the configuration of the SL network

finalized during initialization and onboarding step is also supplied. Afterwards, the SL platform boots-up and initiates the node's connection to the Swarm network, as well as the blockchain network overlay upon the underlying IP network connection between the nodes. The boot-up is an ordered process in which the set of participant nodes designated as peer-discovery nodes (during the initialization phase) are booted-up first, followed by the rest of the nodes in the network.

(3) Integration and training: After integration of the SL API into the common model is agreed upon by all Swarm members, a node joins SL. The model training itself has the following steps:

a. Enrollment: The Swarm Learning training begins with the enrollment in the Swarm smart contract by each node in a one-time process. Each node subsequently records its relevant attributes in the contract such as the uniform resource identifier (URI) from which its own set of trained parameters can be downloaded by other nodes.

b. Local model training: Nodes next proceed to train the local copy of the model iteratively over multiple rounds / epochs. During each epoch, every node trains its local model using one or more data batches for a fixed number of iterations. After the number is reached, it exports the parameter values and shares them to the other nodes and signals the other nodes that it is ready for parameter-sharing.

c. Parameter sharing commences once the number of nodes that are ready for parameter sharing reaches a certain minimum threshold value specified during initialization. The elected epoch leader merges the parameters derived after local training on all nodes after each epoch. The leader uses the URI information of all the participants, to retrieve the parameters from each node to merge the parameters.

d. Parameter merging and update: The SL framework merges the parameters according to the configured merging algorithm (see below) and signals to the other nodes that new parameters are available. Each node then downloads the new parameters from the leader and updates its local model with the new set of parameter values.

e. Stopping criterion check: Finally, the nodes evaluate the model with the updated parameter values using their local data to calculate the validation metrics. The values obtained from this step are shared using the smart contract state. Each node signals to the network that the update and validation step is complete. The leader keeps checking for the update complete

signal from each node. When it discovers that all merge participants have signaled completion, the leader merges the local validation metric numbers to calculate the global metric numbers and marks the synchronization complete.

(4) Testing: After training has been completed the trained model is applied for testing and inference.

An evaluation license for SL can be used for research purposes and initial testing. Such license will also come with the respective instructions for the use of the SL Library so that interested researchers will be able to create the Swarm network environment, including setup of a Swarm node, the Swarm node network, onboarding of Swarm nodes and training rounds within the Swarm network.

Preparation and adaptation of code to be used in a Swarm Learning environment

A Swarm callback is introduced to integrate the model with the SL Library. Minimum number of nodes for synchronization, synchronization interval, validation dataset and batch size are passed as parameters to Swarm callback. The Swarm callback API is

swCallback = SwarmCallback(sync_interval = <number of training batches between syncs>,

min_peers = <minimum peers>,

val_data = <validation dataset>,

val_batch_size = <validation batch size>,

node_weightage = <relative weightage of node’s model weights>)

sync_interval specifies the synchronization interval,

min_peers specifies the minimum number of nodes for model synchronization,

val_data specifies the validation dataset,

val_batch_size specifies the validation batch size,

model_name specifies the name of the model,

node_weightage specifies the relative weightage to be given to model weights of this node

Data visualization

The classification report and confusion matrix were generated with scikit-learn APIs for each permutation. Measurements of sensitivity, specificity, accuracy, and F1 score of each permutation run was read into a table in Excel (Microsoft Excel for Microsoft 365 MSO: Version: 2008 13127.21348, 16.0.13127_21336 64-bit) using Power Query (Microsoft Excel for Microsoft 365 MSO: Version: 2008 13127.21348, 16.0.13127_21336 64-bit) and used for visualization for the different scenarios in Power BI [Version: 2.81.5831.821 64-bit (Mai 2020)] with Box and Whisker chart by MAQ Software (https://appsource.microsoft.com/en-us/product/power-bi-visuals/ WA104381351, version 3.2.1). AUC, positive predictive value, all confidence intervals and statistical tests were calculated using R (version 3.5.2) and the R packages MKmisc (version 1.6) and ROCR (version 1.0.7).

Bibliography

1. Aronson, S. J. & Rehm, H. L. Building the foundation for genomics in precision medicine. *Nature* **526,** 336–342 (2015).

2. *Haendel, M. A., Chute, C. G. & Robinson, P. N. Classification, ontology, and precision medicine. N. Engl. J. Med.* ***379,*** *1452–1462 (2018).*

3. *Warnat-Herresthal, S. et al. Scalable Prediction of Acute Myeloid Leukemia Using High-Dimensional Machine Learning and Blood Transcriptomics. iScience* ***23,*** *100780 (2020).*

4. *Wiens, J. et al. Do no harm: a roadmap for responsible machine learning for health care. Nat. Med.* ***25,*** *1337–1340 (2019).*

5. *Price, W. N. & Cohen, I. G. Privacy in the age of medical big data. Nat. Med.* ***25,*** *37–43 (2019).*

6. *Berlin, D. A., Gulick, R. M. & Martinez, F. J. Severe Covid-19. N. Engl. J. Med.* ***383,*** *2451–2460 (2020).*

7. *Gandhi, R. T., Lynch, J. B. & Del Rio, C. Mild or Moderate Covid-19. N. Engl. J. Med.* ***383,*** *1757–1766 (2020).*

8. *He, J. et al. The practical implementation of artificial intelligence technologies in medicine. Nat. Med.* ***25,*** *30–36 (2019).*

9. *Kels, C. G. HIPAA in the era of data sharing. JAMA* ***323,*** *476–477 (2020).*

10. *McCall, B. What does the GDPR mean for the medical community? Lancet* ***391,*** *1249–1250 (2018).*

11. *Cho, A. AI systems aim to sniff out coronavirus outbreaks. Science* ***368,*** *810–811 (2020).*

12. *Luengo-Oroz, M. et al. Artificial intelligence cooperation to support the global response to COVID-19. Nat. Mach. Intell. (2020). doi:10.1038/s42256-020-0184-3*

13. *Peiffer-Smadja, N. et al. Machine Learning for COVID-19 needs global collaboration and data-sharing. Nat. Mach. Intell. (2020). doi:10.1038/s42256-020-0181-6*

14. *Ge, Y. et al. A data-driven drug repositioning framework discovered a potential therapeutic agent targeting COVID-19. BioRxiv (2020). doi:10.1101/2020.03.11.986836*

15. *Mei, X. et al. Artificial intelligence-enabled rapid diagnosis of patients with COVID-19. Nat. Med.* ***26,*** *1224–1228 (2020).*

16. *Zhang, K. et al. Clinically Applicable AI System for Accurate Diagnosis, Quantitative Measurements, and Prognosis of COVID-19 Pneumonia Using Computed Tomography. Cell* ***182,*** *1360 (2020).*

17. *Council of Europe: Convention for the Protection of Individuals with Regard to Automatic Processing of Personal Data. International Legal Materials* ***20,*** *317–325 (1981).*

18. *LeCun, Y., Bengio, Y. & Hinton, G. Deep learning. Nature* ***521,*** *436–444 (2015).*

19. *Kaissis, G. A., Makowski, M. R., Rückert, D. & Braren, R. F. Secure, privacy-preserving and federated machine learning in medical imaging. Nat. Mach. Intell. (2020). doi:10.1038/s42256-020-0186-1*

20. *Rajkomar, A., Dean, J. & Kohane, I. Machine learning in medicine. N. Engl. J. Med.* ***380,*** *1347–1358 (2019).*

21. *Savage, N. Machine learning: Calculating disease. Nature* ***550,*** *S115–S117 (2017).*

22. *Ping, P., Hermjakob, H., Polson, J. S., Benos, P. V. & Wang, W. Biomedical informatics on the cloud: A treasure hunt for advancing cardiovascular medicine. Circ. Res.* ***122,*** *1290–1301 (2018).*

23. *Char, D. S., Shah, N. H. & Magnus, D. Implementing Machine Learning in Health Care - Addressing Ethical Challenges. N. Engl. J. Med.* ***378,*** *981–983 (2018).*

24. *Finlayson, S. G. et al. Adversarial attacks on medical machine learning. Science* ***363,*** *1287–1289 (2019).*

25. *Konečný, J. et al. Federated Learning: Strategies for Improving Communication Efficiency. arXiv (2016).*

26. *Shokri, R. & Shmatikov, V. Privacy-preserving deep learning. in 2015 53rd Annual Allerton Conference on Communication, Control, and Computing (Allerton) 909–910 (IEEE, 2015). doi:10.1109/ALLERTON.2015.7447103*

27. *Dove, E. S. et al. Genomic cloud computing: legal and ethical points to consider. Eur. J. Hum. Genet.* ***23,*** *1271–1278 (2015).*

28. *Chollet, F. Keras. Github Keras (2015). at <https://github.com/keras-team/keras>*

29. *Zhao, Y. et al. Federated Learning with Non-IID Data. arXiv (2018).*

30. *Leong, S. et al. Existing blood transcriptional classifiers accurately discriminate active tuberculosis from latent infection in individuals from south India. Tuberculosis (Edinb)* ***109,*** *41–51 (2018).*

31. *Zak, D. E. et al. A blood RNA signature for tuberculosis disease risk: a prospective cohort study. Lancet* ***387,*** *2312–2322 (2016).*

32. *Wang, X. et al. ChestX-Ray8: Hospital-Scale Chest X-Ray Database and Benchmarks on Weakly-Supervised Classification and Localization of Common Thorax Diseases. in 2017 IEEE Conference on Computer Vision and Pattern Recognition (CVPR) 3462–3471 (IEEE, 2017). doi:10.1109/CVPR.2017.369*

33. *Corman, V. M. et al. Detection of 2019 novel coronavirus (2019-nCoV) by real-time RT-PCR. Euro Surveill.* ***25,*** *(2020).*

34. *Aschenbrenner, A. C. et al. Disease severity-specific neutrophil signatures in blood transcriptomes stratify COVID-19 patients. Genome Med.* ***13,*** *7 (2021).*

35. *Chaussabel, D. Assessment of immune status using blood transcriptomics and potential implications for global health. Semin. Immunol.* ***27,*** *58–66 (2015).*

36. *Schulte-Schrepping, J. et al. Severe COVID-19 Is Marked by a Dysregulated Myeloid Cell Compartment. Cell* ***182,*** *1419–1440.e23 (2020).*

37. *Esteva, A. et al. Dermatologist-level classification of skin cancer with deep neural networks. Nature* ***542,*** *115–118 (2017).*

38. *Kaissis, G. et al. A machine learning algorithm predicts molecular subtypes in pancreatic ductal adenocarcinoma with differential response to gemcitabine-based versus FOLFIRINOX chemotherapy. PLoS One* ***14,*** *e0218642 (2019).*

39. *Elshafeey, N. et al. Multicenter study demonstrates radiomic features derived from magnetic resonance perfusion images identify pseudoprogression in glioblastoma. Nat. Commun.* ***10,*** *3170 (2019).*

40. *Abadi, M. et al. Deep Learning with Differential Privacy. in Proceedings of the 2016 ACM SIGSAC Conference on Computer and Communications Security - CCS'16 308–318 (ACM Press, 2016). doi:10.1145/2976749.2978318*

41. *Ryffel, T., Dufour-Sans, E., Gay, R., Bach, F. & Pointcheval, D. Partially Encrypted Machine Learning using Functional Encryption. arXiv (2019).*

42. *Salem, M., Taheri, S. & Yuan, J.-S. Utilizing transfer learning and homomorphic encryption in a privacy preserving and secure biometric recognition system. Computers* ***8,*** *3 (2018).*

43. *Kędzior, M. The right to data protection and the COVID-19 pandemic: the European approach. ERA Forum (2020). doi:10.1007/s12027-020-00644-4*
