# Supplementary material for: Swarm Learning for decentralized and confidential clinical machine learning
Source: Nature. 2021 May 26;594(7862):265–70. doi: 10.1038/s41586-021-03583-3 (PMC8189907; doi:10.1038/s41586-021-03583-3)
Supplement: Supplementary file 2 — Reporting Summary [file 41586_2021_3583_MOESM2_ESM.pdf]

nature research

Corresponding author(s): Joachim L. Schultze

Last updated by author(s): Apr 7, 2021

Reporting Summary

Nature Research wishes to improve the reproducibility of the work that we publish. This form provides structure for consistency and transparency in reporting. For further information on Nature Research policies, see our Editorial Policies and the Editorial Policy Checklist.

Statistics

For all statistical analyses, confirm that the following items are present in the figure legend, table legend, main text, or Methods section.

n/a	Confirmed	
☐	☒	The exact sample size (*n*) for each experimental group/condition, given as a discrete number and unit of measurement
☐	☒	A statement on whether measurements were taken from distinct samples or whether the same sample was measured repeatedly
☐	☒	The statistical test(s) used AND whether they are one- or two-sided<br>*Only common tests should be described solely by name; describe more complex techniques in the Methods section.*
☐	☒	A description of all covariates tested
☒	☐	A description of any assumptions or corrections, such as tests of normality and adjustment for multiple comparisons
☐	☒	A full description of the statistical parameters including central tendency (e.g. means) or other basic estimates (e.g. regression coefficient) AND variation (e.g. standard deviation) or associated estimates of uncertainty (e.g. confidence intervals)
☐	☒	For null hypothesis testing, the test statistic (e.g. *F*, *t*, *r*) with confidence intervals, effect sizes, degrees of freedom and *P* value noted<br>*Give P values as exact values whenever suitable.*
☒	☐	For Bayesian analysis, information on the choice of priors and Markov chain Monte Carlo settings
☒	☐	For hierarchical and complex designs, identification of the appropriate level for tests and full reporting of outcomes
☒	☐	Estimates of effect sizes (e.g. Cohen's *d*, Pearson's *r*), indicating how they were calculated

Our web collection on statistics for biologists contains articles on many of the points above.

Software and code

Policy information about availability of computer code

Data collection

Dataset A: All raw data files were downloaded from GEO and the RNA-seq data was preprocessed using the kallisto aligner v.0.43.1 against the human reference genome gencode v27 (GRCh38.p10). For normalization, we considered all platforms independently, meaning that normalization was performed separately for the samples in Dataset A1, A2 and A3, respectively. Microarray data (Datasets A1 and A2) was normalized using the robust multichip average (RMA) expression measures, as implemented in the R package affy (version 1.60.0). RNA-seq data (Dataset A3) was normalized with the R package DESeq2 (version 1.22.2) using standard parameters. In order to keep the datasets comparable, data was filtered for genes annotated in all three datasets, which resulted in 12,708 genes. No filtering of low-expressed genes was performed. All scripts used in this study for pre-processing are provided as a docker container on Docker Hub (version 0.1, https://hub.docker.com/r/schultzelab /aml_classifier) and GitHub (https://github.com/schultzelab/swarm_learning).
Dataset B,D,E: All raw data file were downloaded from GEO or collected at the partner hospitals and aligned to the human reference genome gencode v33 (GRCh38.p13) and quantified transcript counts using STAR v 2.7.3a. For all samples in Datasets B and D,E, raw counts were imported using the R package DESeq2 (version 1.22.2, DESeqDataSetFromMatrix function) and size factors for normalization were calculated using the DESeq function using standard parameters.
Dataset C: The NIH Chest X-Ray dataset was downloaded from https://www.kaggle.com/nih-chest-xrays/data. In order to preprocess the data, we used Python (version 3.6.9) and Keras (version 2.3.1) real-time data augmentation and generation APIs (keras.preprocessing.image.ImageDataGenerator and flow_from_dataframe). The following pre-processing arguments were used: height or width shift range (~ 5%), random rotation range (~ 5 degree), random zoom range (~ 0.15), sample-wise center and standard normalization. Additionally, all images are resized to (128 * 128) from their original size of (1024 * 1024).

Data analysis

All models for the experiments have been implemented using Python (version 3.6.9), Keras (version 2.3.1), Tensorflow (2.2.0-rc2) and scikit-learn (version 0.23.1). The LASSO algorithm has been implemented using Keras (version 2.3.1). All code is available on GitHub (https://github.com/schultzelab/swarm_learning).
Measurements of sensitivity, specificity, accuracy and F1 score of each permutation run was read into a table in Excel (Microsoft Excel for Microsoft 365 MSO: Version: 2008 13127.21348 (16.0.13127_21336 64-bit)) using Power Query (Microsoft Excel for Microsoft 365 MSO: Version: 2008 13127.21348 (16.0.13127_21336 64-bit)) and used for visualization for the different scenarios in Power BI [Version:

2.81.5831.821 64-bit (Mai 2020)] with Box and Whisker chart by MAQ Software (https://appsource.microsoft.com/en-us/product/power-bi-visuals/WA104381351, version 3.2.1).
AUC, positive predictive value, all confidence intervals and statistical tests were calculated using R (version 3.5.2) and the R packages MKmisc (version 1.6) and ROCR (version 1.0.7).

For manuscripts utilizing custom algorithms or software that are central to the research but not yet described in published literature, software must be made available to editors and reviewers. We strongly encourage code deposition in a community repository (e.g. GitHub). See the Nature Research guidelines for submitting code & software for further information.

Data

Policy information about availability of data

All manuscripts must include a data availability statement. This statement should provide the following information, where applicable:

- Accession codes, unique identifiers, or web links for publicly available datasets
- A list of figures that have associated raw data
- A description of any restrictions on data availability

Processed data can be accessed via the SuperSeries GSE122517 or via the individual SubSeries GSE122505 (dataset A1), GSE122511 (dataset A2) and GSE122515 (dataset A3). Dataset B consists of the following series which can be accessed at GEO: GSE101705, GSE107104, GSE112087, GSE128078, GSE66573, GSE79362, GSE84076, and GSE89403. Furthermore, it contains the Rhineland study. This dataset is not publicly available because of data protection regulations. Access to data can be provided to scientists in accordance with the Rhineland Study's Data Use and Access Policy. Requests for further information or to access the Rhineland Study's dataset should be directed to RS-DUAC@dzne.de. Dataset D and E contain dataset B and additional samples for COVID-19. These datasets are made available at the European Genome-Phenome Archive (EGA) under accession number EGAS00001004502 , which is hosted by the EBI and the CRG. The healthy RNA-seq data included from Saarbrücken is available from PPMI through the LONI data archive, https://www.ppmi-info.org/data. The NIH CC Chest X-Ray (Dataset C) can be downloaded from https://www.kaggle.com/nih-chest-xrays/data. Normalized log transformed expression matrices of datasets A1, A2, A3, B, D and E as used for the predictions are made available via FASTGenomics at https://beta.fastgenomics.org/p/swarm-learning.

Field-specific reporting

Please select the one below that is the best fit for your research. If you are not sure, read the appropriate sections before making your selection.

☒ Life sciences ☐ Behavioural & social sciences ☐ Ecological, evolutionary & environmental sciences

For a reference copy of the document with all sections, see nature.com/documents/nr-reporting-summary-flat.pdf

Life sciences study design

All studies must disclose on these points even when the disclosure is negative.

Sample size	For the 12029 samples from data set A (AML), we followed work of Warnat-Herresthal et al, 2020, (doi: 10.1016/j.isci.2019.100780). Dataset B (Tb, 1999 samples) is a collection of all available PAX-based high-quality Tb datasets and controls on GEO. For COVID-19 in dataset D, the collection of 134 samples and 9 controls was driven by availability of consenting patients. For dataset E, the collection of 2400 samples was driven by availability of consenting patients. Dataset C has been compiled and published by the NIH CC and contains 112120 X-ray images. It is one of the largest community data sets and has been used in many studies.
Data exclusions	We used a minimum of five million aligned reads per samples to exclude low-quality samples from the Covid samples. This number is recommended as a minimum for bulk RNA sequencing, as e.g. stated by Illumina (https://support.illumina.com/bulletins/2017/04/considerations-for-rna-seq-read-length-and-coverage-.html)
Replication	The swarm learning approach has been successfully replicated in five data sets (A,B,C,D,E) with multiple permutations.
Randomization	The allocation into experimental group was determined by disease/condition and no other covariates were used. An additional experiment tested the impact of age, sex and COVID-19 diseases severity.
Blinding	Blinding was not applicable, since we collected pre-existing data sets. Additionally to guarantee independent sampling, we performed random permutations of training and test data sets.

Reporting for specific materials, systems and methods

We require information from authors about some types of materials, experimental systems and methods used in many studies. Here, indicate whether each material, system or method listed is relevant to your study. If you are not sure if a list item applies to your research, read the appropriate section before selecting a response.

Materials & experimental systems

n/a	Involved in the study
☒	☐ Antibodies
☒	☐ Eukaryotic cell lines
☒	☐ Palaeontology and archaeology
☒	☐ Animals and other organisms
☐	☒ Human research participants
☒	☐ Clinical data
☒	☐ Dual use research of concern

Methods

n/a	Involved in the study
☒	☐ ChIP-seq
☒	☐ Flow cytometry
☒	☐ MRI-based neuroimaging

Human research participants

Policy information about studies involving human research participants

Population characteristics	The Rhineland Study participants stem from an ongoing community-based cohort study in which all inhabitants of two geographically defined areas in the city of Bonn, Germany aged 30–100 years are being invited to participate. Persons living in these areas are predominantly German with Caucasian ethnicity. Participation in the study is possible by invitation only. The only exclusion criterion is insufficient German language skills to give informed consent.<br>The COVID-19 samples are described in Supplementary Table 6.
Recruitment	The Rhineland Study is an ongoing community-based cohort study in which all inhabitants of two geographically defined areas in the city of Bonn, Germany, aged 30 years and above are being invited to participate. Persons living in these areas are predominantly German from Caucasian descent. Participation in the study is possible by invitation only. The only exclusion criterion is insufficient command of the German language to give informed consent. Therefore, given that participation in the Rhineland Study does not depend on any health-related outcome (e.g. the presence or absence of any particular lifestyle, disease or therapy), the potential risk of any selection bias impacting our results is, in all likelihood, very low.<br>COVID-19 samples were collected based on availability. For all COVID-19 patients, the study was carried out in accordance with the applicable rules concerning the review of research ethics committees and informed consent. All patients or legal representatives were informed about the study details and could decline to participate. COVID-19 was diagnosed by a positive SARS-CoV-2 RT-PCR test in nasopharyngeal or throat swabs and/or by typical chest CT-scan finding.
Ethics oversight	Approval to undertake the Rhineland Study was obtained from the ethics committee of the University of Bonn, Medical Faculty. Collection of Covid19 samples was overseen by the research ethics committees at Radboud University Medical Centre in Nijmegen, the Netherlands (local ethics committee CMO Arnhem-Nijmegen, registration no. 2016-2923), and the Sotiria Athens General Hospital (Ethics Committee of Sotiria Athens General Hospital, IRB 23/12.08.2019) or the ATTIKON University General Hospital ((Ethics Committee of ATTIKON University General Hospital, IRB 26.02.2019) in Athens, Greece as well as the respective committees at the other sites: Kiel, Germany (COVIDOM, Ethics Committee of the University of Kiel, IRB D466/20), Saarbrücken, Germany (CORSAAR, Ethics Committee Medical Association of the Saarland, IRB 62/20, IRB 20200597), Munich, Germany (Ethics Committee of the LMU Munich, IRB 286/2020B01), Tübingen Germany (DeCOI Host Genomes, Ethics Committee of the Medical Faculty of the University of Tübingen, IRB 286/2020B01), Aachen, Germany (COVAS, Ethics Committee of the Medical Faculty of the Technical University Aachen, IRB 20-085), Cologne, Germany (Ethics Committee of the University of Cologne, IRB 20-1187_1) and Bonn, Germany (Ethics Committee of the Medical Faculty of the University of Bonn, IRB 073/19, 134/20). Dataset C is IRB approved (personal communication by Dr. Summers Senior Investigator, Clinical Image Processing Service, NIH CC).

Note that full information on the approval of the study protocol must also be provided in the manuscript.
